# Genetic predisposition to neuroblastoma results from a regulatory polymorphism that promotes the adrenergic cell state

**DOI:** 10.1172/JCI166919

**Published:** 2023-05-15

**Authors:** Nina Weichert-Leahey, Hui Shi, Ting Tao, Derek A. Oldridge, Adam D. Durbin, Brian J. Abraham, Mark W. Zimmerman, Shizhen Zhu, Andrew C. Wood, Deepak Reyon, J. Keith Joung, Richard A. Young, Sharon J. Diskin, John M. Maris, A. Thomas Look

**Affiliations:** 1 Department of Pediatric Oncology, Dana-Farber Cancer Institute, Harvard Medical School, Boston, Massachusetts, USA.; 2 Division of Pediatric Hematology/Oncology, Boston Children’s Hospital, Boston, Massachusetts, USA.; 3 College of Animal Sciences, Zhejiang University, Hangzhou, Zhejiang, China.; 4 National Clinical Research Center for Child Health, National Children’s Regional Medical Center, Children’s Hospital, Zhejiang University School of Medicine, Hangzhou, Zhejiang, China.; 5 Cancer Center, Zhejiang University, Hangzhou, Zhejiang, China.; 6 Department of Pathology and Laboratory Medicine, Children’s Hospital of Philadelphia, Perelman School of Medicine at the University of Pennsylvania, Philadelphia, Pennsylvania, USA.; 7 Department of Oncology and Comprehensive Cancer Center, St. Jude Children’s Research Hospital, Memphis, Tennessee, USA.; 8 Whitehead Institute for Biomedical Research, Cambridge, Massachusetts, USA.; 9 Department of Computational Biology, St. Jude Children’s Research Hospital, Memphis, Tennessee, USA.; 10 Department of Biochemistry and Molecular Biology, Mayo Clinic College of Medicine, Mayo Clinic Cancer Center, Rochester, Minnesota, USA.; 11 Department of Molecular Medicine and Pathology, Faculty of Medical and Health Science, University of Auckland, Auckland, New Zealand.; 12 Molecular Pathology Unit, Center for Computational and Integrative Biology, and Center for Cancer Research, Massachusetts General Hospital, Charlestown, Massachusetts, USA.; 13 Department of Pathology, Harvard Medical School, Boston, Massachusetts, USA.; 14 The Broad Institute of MIT and Harvard, Cambridge, Massachusetts, USA.; 15 Biology Department, MIT, Cambridge, Massachusetts, USA.; 16 Division of Oncology and Center for Childhood Cancer Research, Children’s Hospital of Philadelphia and Perelman School of Medicine at the University of Pennsylvania, Philadelphia, Pennsylvania, USA.

**Keywords:** Genetics, Oncology, Genetic variation, Molecular genetics

## Abstract

Childhood neuroblastomas exhibit plasticity between an undifferentiated neural crest–like mesenchymal cell state and a more differentiated sympathetic adrenergic cell state. These cell states are governed by autoregulatory transcriptional loops called core regulatory circuitries (CRCs), which drive the early development of sympathetic neuronal progenitors from migratory neural crest cells during embryogenesis. The adrenergic cell identity of neuroblastoma requires LMO1 as a transcriptional cofactor. Both *LMO1* expression levels and the risk of developing neuroblastoma in children are associated with a single nucleotide polymorphism, G/T, that affects a GATA motif in the first intron of *LMO1*. Here, we showed that WT zebrafish with the GATA genotype developed adrenergic neuroblastoma, while knock-in of the protective TATA allele at this locus reduced the penetrance of MYCN-driven tumors, which were restricted to the mesenchymal cell state. Whole genome sequencing of childhood neuroblastomas demonstrated that TATA/TATA tumors also exhibited a mesenchymal cell state and were low risk at diagnosis. Thus, conversion of the regulatory GATA to a TATA allele in the first intron of *LMO1* reduced the neuroblastoma-initiation rate by preventing formation of the adrenergic cell state. This mechanism was conserved over 400 million years of evolution, separating zebrafish and humans.

## Introduction

Across all cell lineages and tissue types, groups of transcription factors act cooperatively in autoregulatory loops to bind to their own and each other’s enhancers to regulate gene expression, forming core regulatory circuitries (CRCs). The transcription factors of the CRCs also bind coordinately to regulate the expression of downstream genes across each circuit’s extended regulatory network, thereby governing cell identity and lineage specification (1). Arendt et al. (2) have proposed that conservation of cell-type identity across species reflects evolutionarily determined composition of the transcription factors that define key CRCs, leading to orderly progression of conserved lineage-specific gene expression. Across vertebrates, a set of neural crest–specific transcription factors facilitate the development of the neural crest, which gives rise to a wide spectrum of diverse cell lineages, including neurons of the peripheral nervous system, glia, melanocytes, and facial bones and cartilage (3). In neuroblastoma — a pediatric malignancy originating from neural crest–derived progenitor cells — neuroblasts fail to differentiate while transforming to a malignant cell state, often driven by aberrant high-level expression of *MYCN* or *MYC* (4, 5).

There are at least 2 primary CRC transcriptional networks in neuroblastoma that drive the growth and survival of neuroblastoma tumors. The most prevalent is the adrenergic CRC, including HAND2, GATA3, ISL1, PHOX2B, TBX2, ASCL1, and TFAP2B — which is associated with committed progenitors of the sympathoadrenal cell lineage — while the mesenchymal CRC, including a large number of candidate master transcription factors such as NOTCH2, BACH1, ID1, EGR3, FLI1, CBFB, and STAT3, represents a less differentiated mesenchymal or neural crest-cell–like transcriptional cell state (6–9). Interestingly, neuroblastoma tumors are often composed of both adrenergic and mesenchymal tumor cell populations that may spontaneously interconvert between networks, at least in part due to activation of the NOTCH pathway, which drives the mesenchymal cell state (10, 11). Therapeutic strategies are being designed to target transcription factors within the adrenergic and mesenchymal CRCs and their downstream extended networks of genes and pathways, which may prove useful for targeting tumor cells dependent on these transcriptional networks for growth and survival in the future (7, 10, 12).

LIM-domain-only 1 (LMO1) acts an essential transcriptional coregulator for the formation of the adrenergic neuroblastoma CRC (13). However, it is not part of the CRC itself because it is not a transcription factor and lacks a DNA binding domain. LMO1 facilitates transcription as a bridge protein by forming protein-protein interactions through its 2 zinc-finger LIM domains (14, 15). We have demonstrated that LMO1 overexpression synergizes with MYCN to accelerate tumor onset, penetrance, and metastasis in a *dβh:MYCN*- driven zebrafish model (16).

Our GWAS identified multiple SNPs in noncoding sequences across the genome that are associated with neuroblastoma susceptibility (17–20). In an earlier publication, we showed that one of the most significant neuroblastoma predisposition loci in our study was the rs2168101 G **→** T transversion that resides within the first intron of the *LMO1* gene (20). We showed that the G-containing allele of this SNP represents the permissive allele associated with increased risk of developing neuroblastoma, while the T-containing allele is protective of developing neuroblastoma (20).

The T-containing allele of rs2168101 has been reported to be much more common in European and East Asian populations, with an allele frequency of approximately 30%, while it is rare or even absent in African populations, suggesting that the T-containing allele arose in human populations as a single mutational event, concurrent with, or shortly after, migration out of Africa (20). By contrast, the reported T-containing allele frequency in European and European American patients with neuroblastoma ranged from 16%–24%, depending on cohort (20), which was significantly lower than one would expect based on the allelic frequency in healthy controls, indicating that the T-containing allele inhibited the development of neuroblastoma. Since the original GWAS findings reported by Oldridge et al. in 2015, other studies have confirmed the protective role of the T-containing allele in neuroblastoma (21, 22). A metaanalysis of published literature on neuroblastoma susceptibility loci conducted by Hashemi et al. summarizes the 3 published research studies on rs2168101 and provides a combined odds ratio of 0.39 of developing neuroblastoma in patients with the TATA/TATA genotype compared with the GATA/GATA genotypes, demonstrating a significantly lower risk of developing neuroblastoma in patients with the T-containing allele (23). The mechanistic basis underpinning this observation is the focus of this report.

In our current study, we used genetic editing in the zebrafish to define the in vivo mechanism underlying the striking association between the germline rs2168101 G **→** T noncoding polymorphism in the first intron of *lmo1* and the risk of developing neuroblastoma. We found that the G-containing allele is essential for the formation of the adrenergic CRC in humans and zebrafish, so that people and fish with the T-containing allele at this position only develop tumors relying on the mesenchymal CRC, which is much less efficient in initiating neuroblastoma.

## Results

### The rs2168101 locus is highly conserved throughout evolution.

**Figure 1:**
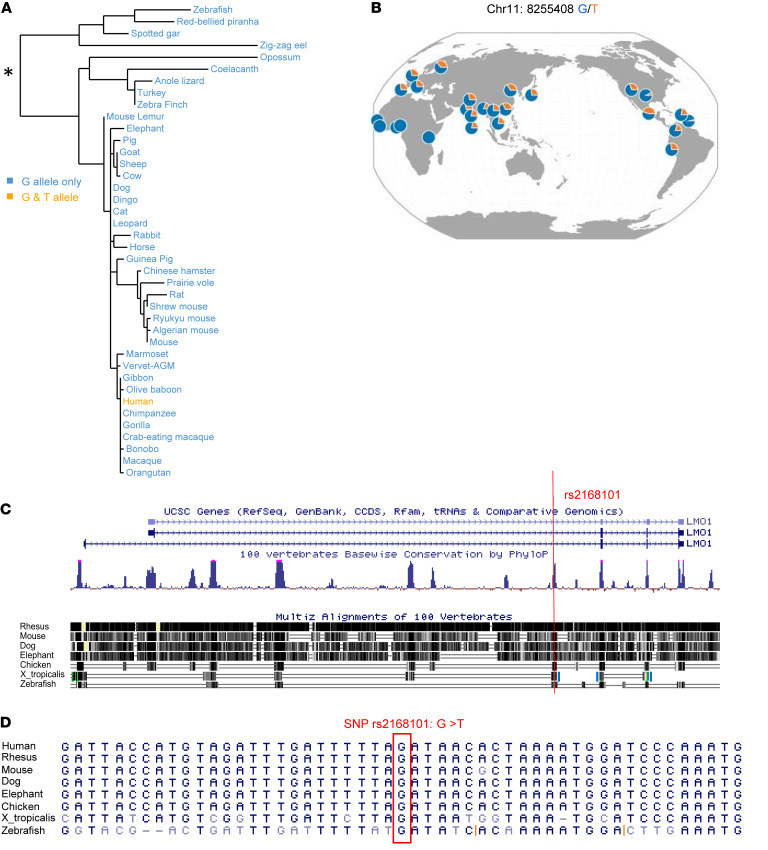
Evolutionary history of the G/T polymorphism at rs2168101. (**A**) Phylogenetic tree representing the evolutionary relationship between the *LMO1* genes from the indicated species over the last 400 million years. Blue font denotes species that exclusively harbor a G at rs2168101. Orange font denotes that only humans demonstrate a G/T polymorphism at the rs2168101 locus. (**B**) Distribution of the G and T alleles of rs2168101 across Human Genome Diversity Project (HGDP) populations, as illustrated by their genome browser (http://popgen.uchicago.edu/ggv/). Circles create a pie chart in which blue represents the proportion of human populations from different parts of the world with a G at rs2168101 (human chromosome 11, position 8255408), and orange represents the proportion with a T. (**C**) Shown is a modified UCSC Genome Browser (https://genome.ucsc.edu/) window of the human *LMO1* locus indicating the 2 alternative transcription start sites and the position of rs2168101 in the first intron (top), a vertebrate conservation track graphing PhyloP conservation scores (middle), and Multiz alignments of multiple vertebrate species (bottom), illustrating a high level of conservation in the noncoding region surrounding rs2168101. (**D**) The immediate sequence neighborhood surrounding rs2168101 in the first intron of *LMO1* from multiple species is shown. The G at rs2168101 in the human consensus sequence is marked with a red box.

Previously, we showed that the human G **→** T polymorphism at the rs2168101 locus within the first intron of the *LMO1* gene comprised either a guanine (G) — coding for a permissive allele associated with increased risk of developing neuroblastoma — or a thymine (T) — coding for an allele protective of the development of neuroblastoma (20). The evolutionary history of the G **→** T polymorphism is shown in Figure 1A, which illustrates the finding that the G-containing allele is exclusively found at this position throughout evolution, with the exception of human populations, which are the first to have the T-containing allele. Interestingly, the T-containing allele does not even appear in highly related nonhuman primates such as gorillas and orangutans (Supplemental Figure 1; supplemental material available online with this article; https://doi.org/10.1172/JCI166919DS1). The reference G-containing allele, by contrast, can be tracked back at least 400 million years to osteichthyes, the common ancestor of zebrafish and humans (Figure 1A). Interestingly, the T-containing allele at rs2168101 in humans is well represented at comparable frequencies in European, Asian, and American populations, whereas it is nearly absent in Africans (Figure 1B and Supplemental Figure 2), suggesting that the T-containing allele arose in human populations as a single mutational event around the time of human migration out of Africa.

Most noncoding intronic sequences within the *lmo1* gene are not well conserved between zebrafish and humans. The few exceptions are regions that consist of a few hundred base pairs of highly conserved sequence, including the region containing the rs2168101 locus, which likely contain important regulatory motifs (Figure 1C). We found that the noncoding region surrounding the conserved G-containing allele at rs2168101 is highly conserved (Figure 1, C and D), and 73% of the flanking 20 bp on each side of the G-containing allele are identical between human and zebrafish (30 bp out of 41 bp) (Figure 1D). The *lmo1* coding sequence is also highly conserved among vertebrates (Figure 1C), with 84.5% (398/471) nucleotide identity between human and zebrafish and 98% identity at the amino acid level, including 2 perfectly identical LIM domains (Supplemental Figure 4D). These data, in combination with our previous studies, are consistent with a role for the G-containing allele as part of a highly conserved regulatory element controlling *LMO1* expression in the developing parasympathetic nervous system (PSNS) (20).

### Substitution of a T-containing allele for the G-containing allele at rs2168101 impairs the initiation of neuroblastoma in a MYCN-driven zebrafish model.

**Figure 2:**
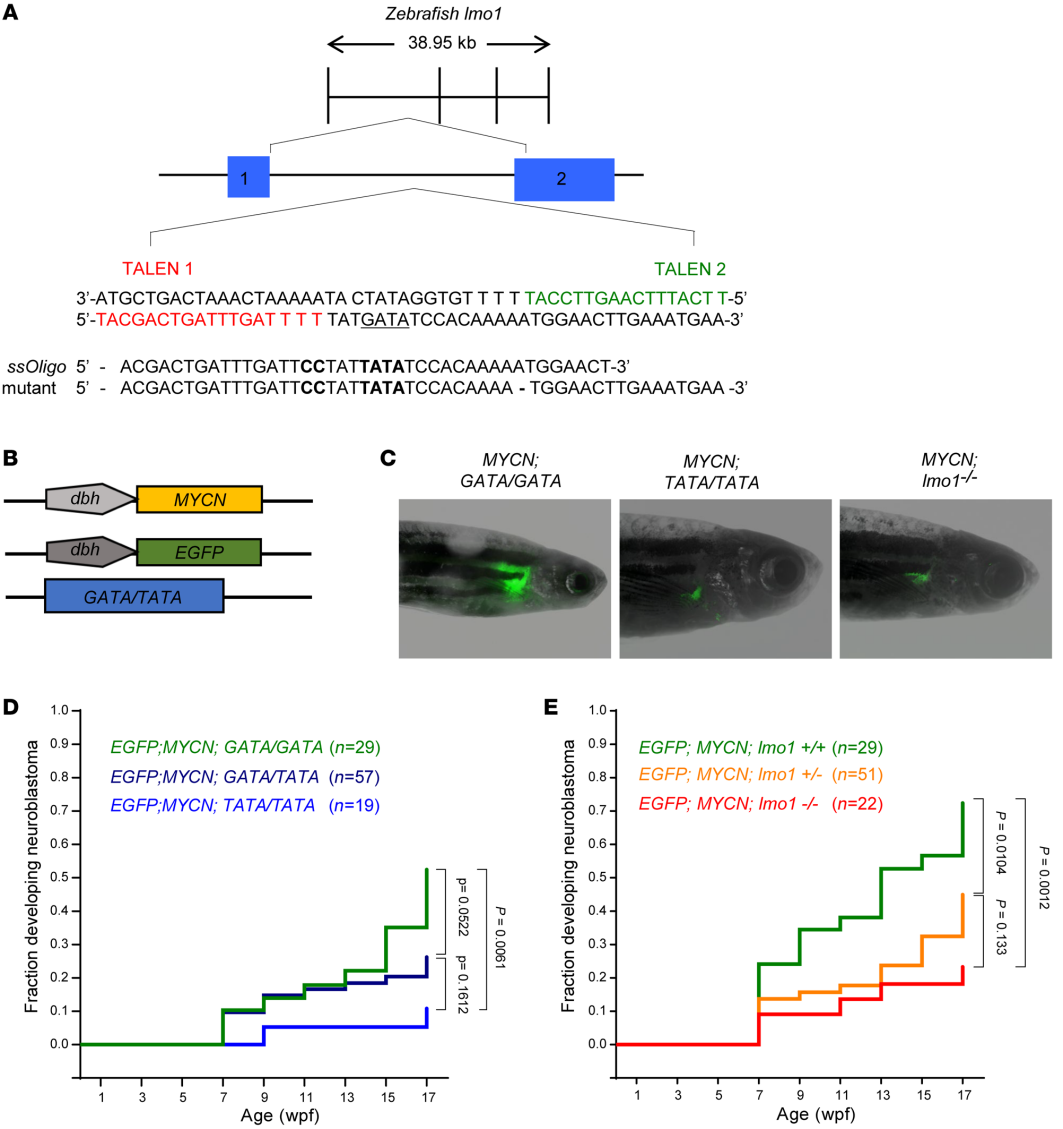
The development of *MYCN*-driven neuroblastomas is impaired in the *TATA/TATA* and *lmo1*-null backgrounds. (**A**) Diagram illustrating the construction of the *lmo1 GATA/TATA* (*GATA/TATA*) zebrafish line in which TALEN-mediated gene editing was used to replace the G at rs2168101 with a T. The rs2168101 G resides within the first intron of the zebrafish *lmo1* gene (exons 1 and 2 are denoted by blue boxes) and creates the first nucleotide of a *GATA* DNA-binding sequence (in bold). To facilitate the precise genome editing and knock-in of the T allele at this locus, we used TALEN gene-editing technology targeting the sequences flanking the G at rs2168101 (as indicated in red and green) together with a single-stranded DNA oligonucleotide containing a T instead of the G with short flanking homology arms of 20 nucleotides (*TATA-ssOligo*). To prevent TALEN binding to the 5′ arm and activity after successful knock-in of the *TATA-ssOligo* and to aid in the identification of embryos containing the modified sequence, the *TATA-ssOligo* was designed with 2 additional nucleotide changes (CC to replace TT in the 5′ homology arm, marked in bold) to create a new restriction site for TfiI (see also Supplemental Figure 3). (**B**) To analyze the effect of the rs2168101 G **→** T substitution on *MYCN*-induced neuroblastoma, compound transgenic zebrafish lines were created by crossing the transgenic lines *Tg(dβh:MYCN) and Tg(dβh:EGFP)* with the *GATA/TATA* knock-in line, as illustrated. The *dβh:EGFP* and *dβh:MYCN* lines, in which the zebrafish *dβh* promoter was used to facilitate tissue-specific expression of *EGFP and MYCN,* were established previously (24). (**C**) Representative fluorescent images of adult zebrafish showing EGFP-expressing tumors arising in the indicated transgenic lines. (**D** and **E**) Starting at 5 weeks postfertilization (wpf), zebrafish with the indicated genotypes were monitored biweekly for the presence of tumors by EGFP fluorescence microscopy. The graph shows a Kaplan-Meier analysis of the cumulative frequency of neuroblastomas in the transgenic lines. Statistical analysis was performed using the logrank test.

To dissect the mechanism through which the T-containing allele at rs2168101 protects children from developing neuroblastoma, we used transcription activator–like effector nuclease–mediated (TALEN-mediated) gene editing to generate the rs2168101 T-containing allele in zebrafish (Figure 2A and Supplemental Figure 3; see Methods for details). Because the G at rs2168101 comprises the first nucleotide of a highly conserved *GATA* site, we designated the heterozygous mutant line *lmo1 GATA/TATA* (also referred to herein as the *GATA/TATA* line). We found that the heterozygous *GATA/TATA* and homozygous *TATA/TATA* fish were viable, developmentally normal, and fertile. We crossed the *GATA/TATA* line with our previously described *Tg(dβh:MYCN;dβh:EGFP)* transgenic zebrafish line — in which *MYCN* and *EGFP* expression are driven in the PSNS by the dopamine β-hydroxylase promoter — and *MYCN* overexpression gave rise to neuroblastoma in the inter-renal gland (IRG) (Figure 2, B and C) (24). The IRG is the zebrafish equivalent of the adrenal medulla, which is the most frequent site of human neuroblastoma (4, 24). We have shown that the tumors that arise in the IRG in this transgenic fish model are small, round, blue cell tumors expressing tyrosine hydroxylase and synaptophysin, which are markers of human neuroblastoma (24).

Three different genetically modified zebrafish lines were generated to determine the influence of the *TATA/GATA* regulatory site on the rate of initiation and penetrance of neuroblastoma: *Tg(dβh:MYCN; dβh:EGFP; GATA/GATA*), *Tg(dβh:MYCN; dβh:EGFP; GATA/TATA*), and *Tg(dβh:MYCN; dβh:EGFP; TATA/TATA*). We monitored offspring for the onset of EGFP^+^ tumor masses in the anterior region of the abdomen where the IRG is located (Figure 2C). Although tumors arose more frequently in the GATA/GATA than in the GATA/TATA genotype (Figure 2D), the overall tumor onset curves for *GATA/GATA* fish and *GATA/TATA* fish were not statistically significantly different, suggesting that, in this model, 1 intact GATA site was sufficient to promote neuroblastoma. By contrast, only 10% of the *TATA/TATA* fish developed neuroblastoma over the first 17 weeks of life, which represented a significantly lower penetrance than the tumor onset for the *GATA/GATA* fish (*P <* 0.01). This finding is consistent with the significant overrepresentation of homozygous *GATA/GATA* genotypes in neuroblastoma revealed by GWAS in children, in that 57.9% of children with neuroblastoma had GATA/GATA, compared with 35.6% for *GATA/TATA*, and only 6.4% for the *TATA/TATA* genotype (20). Thus, our studies in the zebrafish model are very consistent with GWAS findings in children, demonstrating that the G-containing allele at rs2168101 increases the risk of developing neuroblastoma, while the T-containing allele at this position is protective.

### Knockout of lmo1 in zebrafish reduces the penetrance of MYCN-induced neuroblastoma.

To independently test whether complete loss of *lmo1* expression confers protection against the development of neuroblastoma, we used CRISPR-Cas9–mediated gene editing to generate a *lmo1* knockout allele containing a 32 bp deletion of coding sequences in the second exon that led to premature termination of translation (Supplemental Figure 4). As previously reported in an *Lmo1* mouse knockout model (25), we found that zebrafish with homozygous knockout of the *lmo1* gene were viable, developmentally normal, and fertile. The *lmo1^+/–^* line was bred with the *Tg(dβh:MYCN; dβh:EGFP)* line and then the *lmo1*^+/–^ progeny were inbred to generate zebrafish with the following genotypes for analysis: *Tg(dβh:MYCN; dβh:EGFP; lmo1^+/+^* ), *Tg(dβh:MYCN; dβh:EGFP; lmo1^+/–^*), and *Tg(dβh:MYCN; dβh:EGFP; lmo1^–/–^*). Analysis of fish with these 3 genotypes showed a significantly reduced penetrance of neuroblastoma at 17 weeks of age in *lmo1*^–/–^ compared with *lmo1^+/+^* zebrafish (22% versus 70% respectively; *P <* 0.01) (Figure 2E). The heterozygous *lmo1^+/–^* fish showed an intermediate tumor penetrance, with 41% of fish developing tumors at 17 weeks. Thus, the protective effect of complete loss of expression of a functional *lmo1* gene (Figure 2E) was similar to the effect of the G **→** T substitution at rs2168101 (Figure 2D). These data suggest that increased levels of lmo1 expression in neuronal progenitor cells most likely account for the increase in neuroblastoma penetrance associated with the G-containing allele at rs2168101.

### Other Lim-only protein family members generally don’t compensate for diminished lmo1 expression in zebrafish MYCN-driven neuroblastomas.

**Figure 3:**
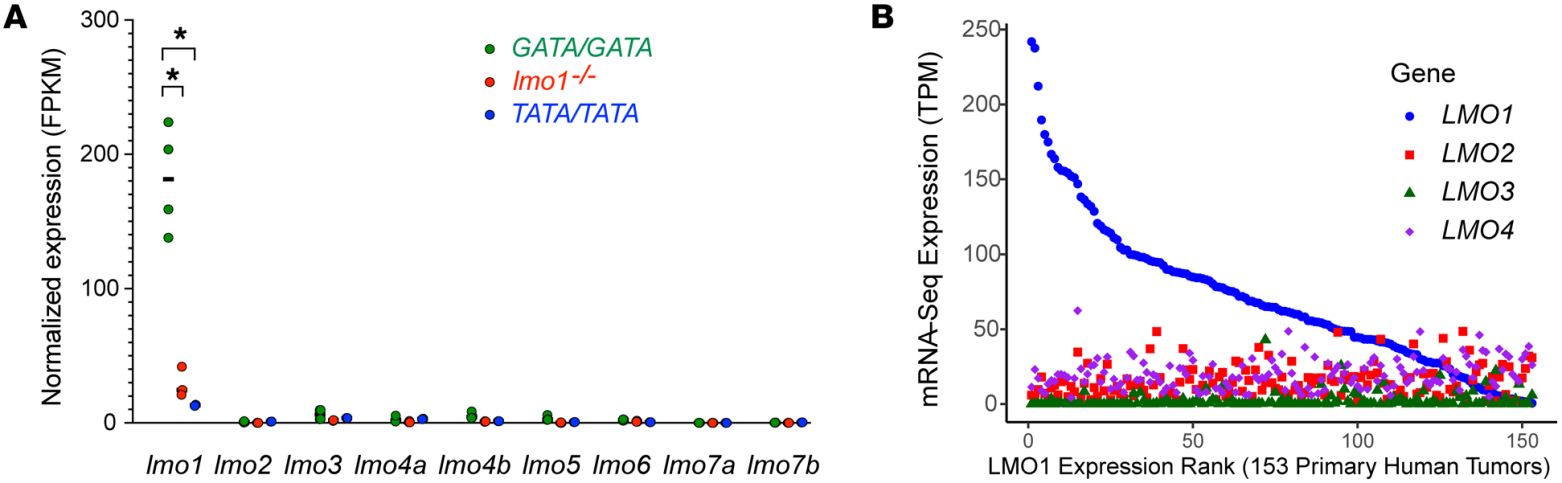
Expression of the *lmo* family genes in zebrafish and human neuroblastoma. (**A**) RNA-Seq analysis was performed to measure the relative mRNA expression of *lmo* family genes in neuroblastomas arising in zebrafish with the indicated genotypes (*GATA/GATA*, *n* = 4; *lmo1^–/–^*, *n* = 3; and *TATA/TATA*, *n* = 2). mRNA expression levels for the indicated *lmo* family genes are represented by FPKM log-scale values. Expression under 1 FPKM is considered as nonexpressed. Statistical analysis was performed using the 2-tailed, unpaired *t* test. *P < 0.005. (**B**) Relative *LMO1-4* mRNA expression levels measured by RNA-Seq in 153 primary human neuroblastoma samples (from TARGET), ranked from highest (left) to lowest levels of *LMO1* expression (in FPKM). Expression correlation analysis demonstrated weak inverse correlation between LMO1 and the other 3 LMO family members (R ≥ –0.3, *P <* 0.05).

*LMO1, LMO2,* and *LMO3* are functionally redundant T-ALL oncogenes (26–28), so, we reasoned that loss of *lmo1* expression in zebrafish might stimulate the upregulation of other *lmo* family members during neuroblastoma pathogenesis. We therefore analyzed gene expression in *MYCN*-induced neuroblastomas in *GATA/GATA*, *TATA/TATA* and *lmo1*^–/–^ genetic backgrounds by RNA-Seq. Consistent with human neuroblastomas (20), *lmo1* mRNA expression levels in neuroblastomas from *GATA/GATA* fish were significantly higher than in tumors from either *TATA/TATA* or *lmo1*^–/–^ fish. The *lmo1*^–/–^ fish were included in this study as a control group lacking intact lmo1 protein expression, and the lower lmo1 mRNA levels in these fish are likely due to nonsense-mediated degradation of the mutant lmo1 mRNA containing a premature stop codon in exon 2 (Figure 3A and Supplemental Figure 4). Importantly, expression of the other 6 highly related zebrafish *lmo* family members — *lmo2, lmo3, lmo4a, lmo4b, lmo5, lmo6, lmo7a,* and *lmo7b* — was much lower than *lmo1* in the *GATA/GATA* fish and showed no appreciable differences between the 3 tumor genotypes (Figure 3A). Thus, there was no obvious compensatory overexpression of other *lmo* family members due to the low levels of *lmo1* mRNA expression in zebrafish *TATA/TATA* tumors.

Similarly, analysis of 153 primary human neuroblastoma samples by RNA-Seq showed a weak inverse correlation between *LMO1* expression and the expression levels of the other *LMO* family members (R ≥ –0.3, *P <* 0.05) (Figure 3B). The weakness of this correlation is illustrated by the fact that the strongest inverse correlation was observed between *LMO1* and *LMO3* expression levels (R= –0.3), meaning that only about 10% (R^2^ ≈ 0.1) of the *LMO3* expression level can be explained by low *LMO1* expression in human neuroblastoma samples. Since LMO1 has a central role as a transcriptional cofactor in the adrenergic neuroblastoma CRC (13)*,* the lack of compensation by other *lmo* family members in zebrafish tumors with low *lmo1* expression in *MYCN*-driven neuroblastomas suggests that neuroblastomas arising in fish with the *TATA/TATA* and *lmo1*^–/–^ genotypes employ mechanisms of transformation independent of lmo-family proteins.

### Lmo1 coregulates the adrenergic neuroblastoma CRC.

LIM-only proteins are well known to function as “linker” proteins that facilitate the assembly of transcription factor complexes involved in tissue-specific gene regulation (29–32). We have shown that LMO1 functions in this capacity as an essential transcriptional cofactor for the human adrenergic neuroblastoma CRC (13). To analyze the neuroblastoma CRC in *MYCN*-driven tumors with disrupted *lmo1* expression, we dissected the EGFP-fluorescent tumors from juvenile zebrafish, mechanically prepared a single-cell suspension, sorted the EGFP^+^ cells before RNA extraction, and conducted RNA-Seq to compare the transcriptomes of zebrafish *MYCN*-driven neuroblastomas with the *GATA/GATA*, *TATA/TATA* and *lmo1*^–/–^ lines.

**Figure 4:**
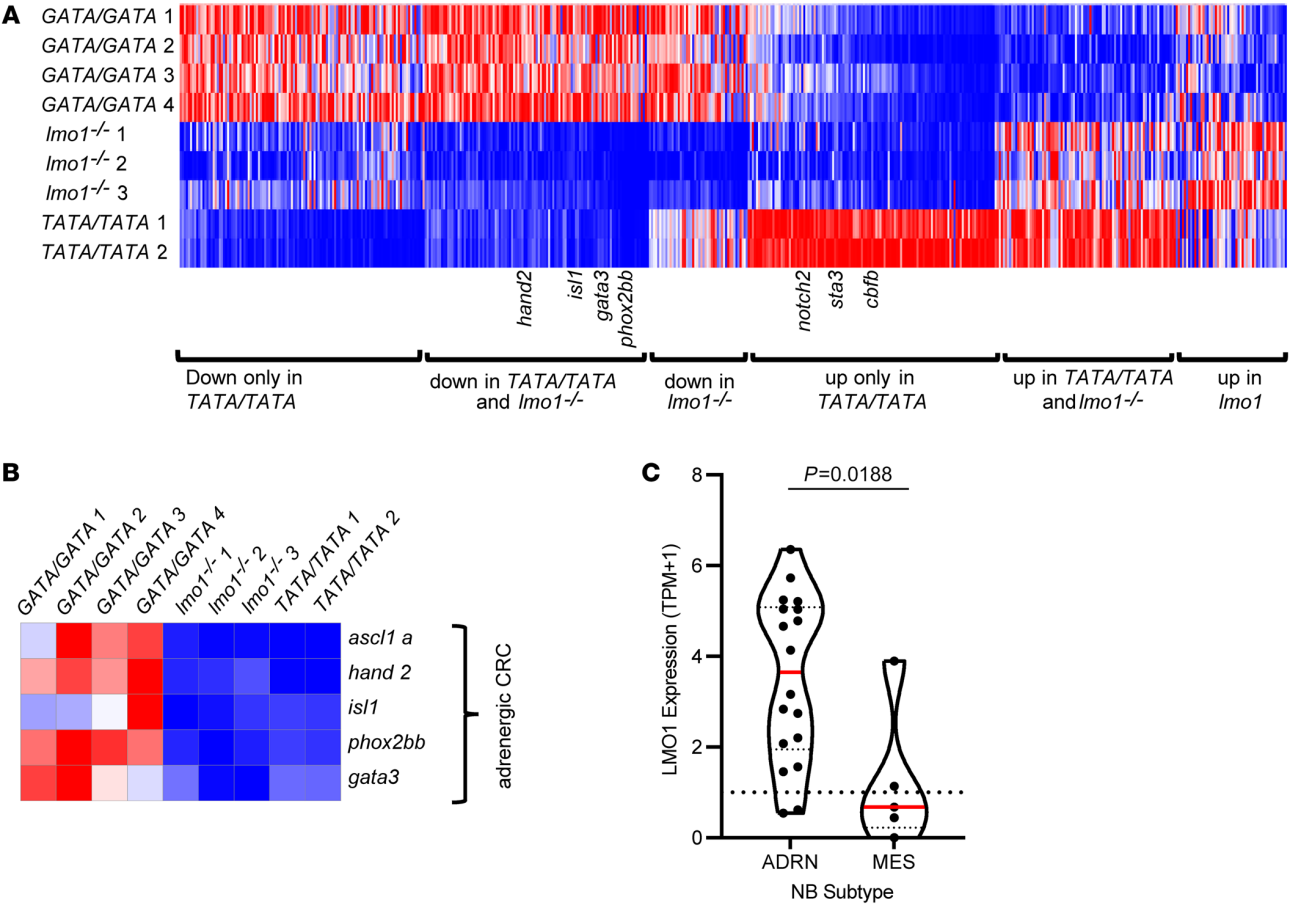
*Lmo1* coregulates transcription factors that comprise the adrenergic neuroblastoma CRC. (**A**) Heatmap image based on RNA-Seq data analysis showing differentially expressed genes in *MYCN*-induced neuroblastoma tumors arising in *lmo1 GATA/GATA* (WT), *lmo1*^–/–^ and *lmo1 TATA/TATA* backgrounds categorized into 6 groups, as indicated. Each row corresponds to a gene, and signal intensity is normalized across the row. Genes were rank ordered from highest (right side of the map) to lowest (left side of the map) based on fold change of gene expression in *TATA/TATA* or *lmo1*^–/–^ compared to *GATA/GATA*. (**B**) Heatmap representing gene expression changes of the known adrenergic neuroblastoma CRC transcription factors *isl1, gata3, ascl1, phox2b,* and *hand2* in *MYCN*-induced neuroblastoma tumors arising in the *GATA/GATA, lmo1*^–/–^ and *TATA/TATA* backgrounds. (**C**) *LMO1* mRNA expression (TPM+1) violin plots retrieved from the 21Q1 release of Depmap (depmap.org) (18), from 23 neuroblastoma cell lines. Cell lines were defined as adrenergic (ADRN) (*n* = 18) or mesenchymal (MES) (*n* = 5) subtypes based on general gene expression profiles. Red bars indicate mean, dotted line indicates a TPM+1 of 1. *P* = 0.0188 by student’s *t* test.

Figure 4A provides an overview of significant differences in gene expression of zebrafish tumor cells in the *GATA/GATA, TATA/TATA* or the lmo1^–/–^ genetic backgrounds (*P* < 0.05 based on an absolute log_2_ fold change compared with GATA/GATA of ≥ 0.45). Ninety transcription factor genes were expressed significantly lower in neuroblastomas from both *TATA/TATA* and *lmo1*^–/–^ zebrafish than in tumors in the GATA/GATA background. These differentially expressed transcription factors include *lmo1,* as well as *gata3*, *hand2*, *phox2bb*, *isl1*, and *ascl1*, which form the adrenergic CRC of neuroblastoma (Figure 4B) (7, 13). In addition, *LMO1* expression levels are also higher in human neuroblastoma cell lines of the adrenergic subtype and lower in the mesenchymal type (Figure 4C and Supplemental Table 2), consistent with our recent study showing that LMO1 is an essential transcriptional cofactor of the adrenergic CRC (13). These results indicate that high levels of *lmo1* expression by the neuroblastoma cells are required to establish the adrenergic cell state.

### Zebrafish TATA/TATA neuroblastomas adopt the mesenchymal CRC.

**Figure 5:**
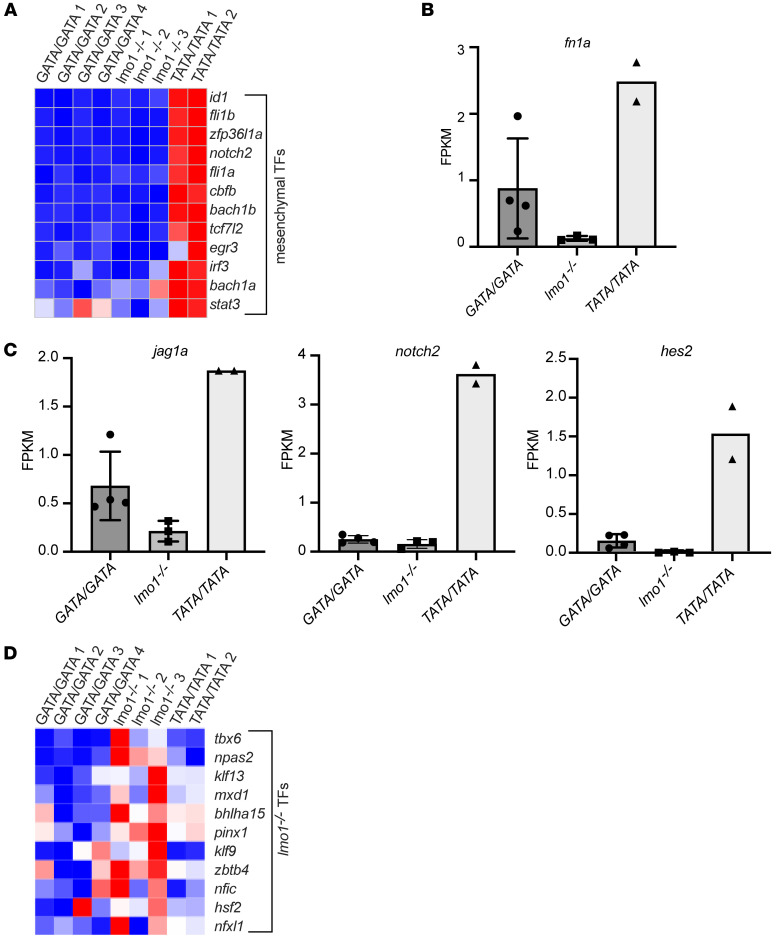
*MYCN*-driven neuroblastomas from the *TATA/TATA* line, but not the *lmo1^–/–^* line, express the mesenchymal CRC. (**A**) Heatmap image based on RNA-Seq data analysis showing the indicated genes of the mesenchymal CRC, which are upregulated in the *MYCN*-induced neuroblastomas arising in the *TATA/TATA* background, but not in the *lmo1*^–/–^ background, compared with the *GATA/GATA* zebrafish. (**B**) Relative mRNA expression levels of the mesenchymal neuroblastoma phenotype marker *fn1a* in neuroblastoma arising in zebrafish with the indicated genotypes (*GATA/GATA*, *n* = 4; *lmo1^–/–^*, *n* = 3; and *TATA/TATA*, *n* = 2). (**C**) Relative mRNA expression levels of NOTCH pathway genes, including *jag1a*, *notch2*, and *hes2*, in neuroblastomas arising in zebrafish from the indicated genotypes (*GATA/GATA*, *n* = 4; *lmo1^–/–^*, *n* = 3; and *TATA/TATA*, *n* = 2). (**D**) Heatmap of transcription factors exclusively upregulated in *MYCN*-driven neuroblastomas from the *lmo1^–/–^* line. TFs; transcription factors.

Because neither *lmo1*^–/–^ nor *TATA/TATA* *MYCN*–expressing tumors appeared to be driven by the adrenergic CRC, which is preferred by neuroblastomas arising in the GATA/GATA background, we questioned whether either tumor type was instead driven by the mesenchymal CRC (6, 7, 10). Further analysis of the RNA-Seq data revealed that mesenchymal neuroblastoma CRC transcription factors, including *notch2*, *id1*, *egr3*, *irf3*, *cbfb, bach1a, bach1b, tcf7l2,* and *mef2b* (6), were upregulated in the *TATA/TATA* tumors compared with *GATA/GATA*, but not in the *lmo1*^–/–^ tumors (Figure 5A). Consistent with these findings, *TATA/TATA* tumors also showed upregulation of the mesenchymal marker fibronectin 1a (*fn1a;* Figure 5B) (6).

Because the NOTCH pathway was shown to reprogram adrenergic neuroblastoma cells to adopt a mesenchymal cell state (10), we examined the RNA-Seq data for genes associated with NOTCH pathway upregulation. Indeed, *TATA/TATA* tumors, but not *lmo1*^–/–^ tumors, showed upregulation of notch receptor (*notch2b*) and ligand (*jagged1*) genes, as well as the NOTCH target gene *hes2* (Figure 5C). Interestingly, the *lmo1*^–/–^ tumors did not highly express the signature genes from either the adrenergic or mesenchymal cell states (Figure 4B and Figure 5A), but instead expressed an alternative set of transcription factors with no previously defined role in either of these 2 neuroblastoma CRCs (Figure 5D).

### Human TATA/TATA neuroblastomas resemble zebrafish TATA/TATA tumors with low LMO1 expression and a mesenchymal expression profile.

**Figure 6:**
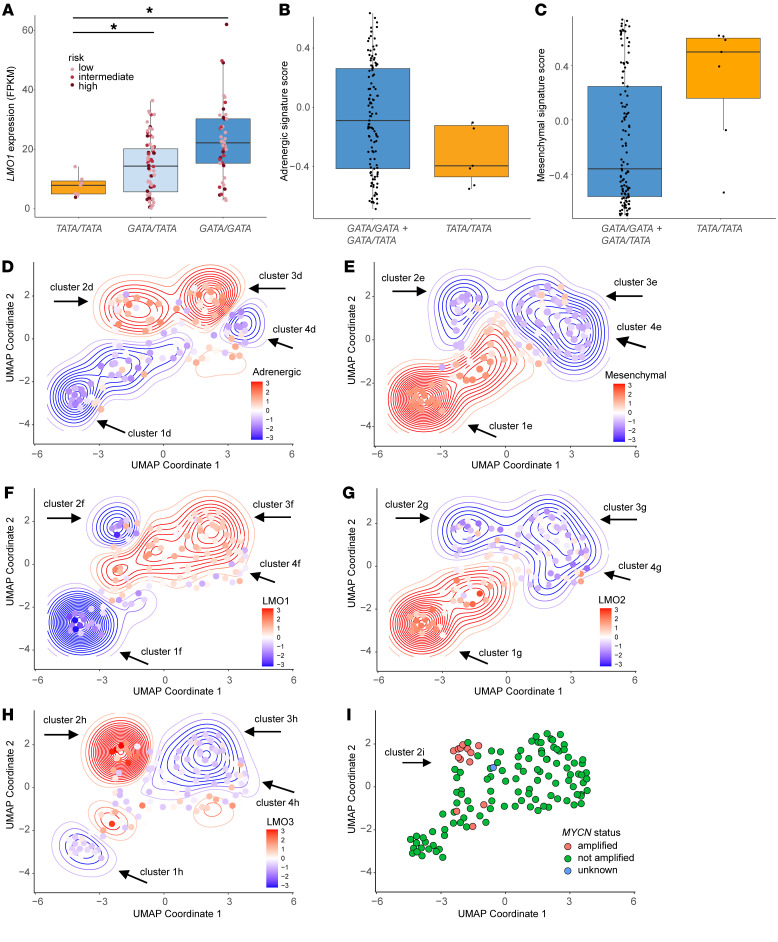
Human *TATA/TATA* neuroblastomas resemble zebrafish *TATA/TATA* tumors with low *LMO1* expression and a mesenchymal phenotype. (**A**) Relative LMO1 mRNA expression levels (in FPKM) in 124 human neuroblastoma samples with the indicated genotypes at rs2168101: TATA/TATA, GATA/TATA, or GATA/GATA. Each neuroblastoma sample is assigned to either low (pink), intermediate (light red) or high risk (purple). Statistical analysis was performed using the 2-tailed, Welch’s *t* test. *P < 0.005. (**B** and **C**) Adrenergic and mesenchymal gene set signature scores were generated using GSVA based on the previously published expression profiles by Von Groningen, et al. (8). Higher positive scores indicate upregulation of the corresponding signature, whereas, lower negative scores indicate downregulation. GATA/GATA and GATA/TATA tumors were combined into 1 group (blue) and compared with the TATA/TATA tumors (tan). (**D** and **E**) UMAP representing the whole transcriptome landscape of 124 human neuroblastoma samples (dots) in a 2-dimensional space combined with the adrenergic (**D**) and mesenchymal (**E**) gene signatures. Relative Z-score-transformed expression for each signature (according to the heat scale) is shown for each tumor (points) and overall density (contours). Clusters 1–4 represent tumors falling into similar density contours based on the adrenergic signature (**D**) or mesenchymal signature (**E**). (**F**–**H**) Combination of UMAP dimensionality reduction analysis and expression levels of *LMO1* (**F**), *LMO2* (**G**), and *LMO3* (**H**). Relative Z-score of log_2_-transformed expression for *LMO1* (**F**), *LMO2,* (**G**) or *LMO3* (**H**) (according to the heat scale) is shown for each tumor (points) and overall density (contours). (**I**) MYCN status for all 124 human neuroblastoma samples.

To test the hypothesis that human neuroblastomas arising in the *TATA/TATA* background have low *LMO1* expression and, as in the zebrafish model, are enriched for neuroblastomas relying on the mesenchymal rather than the adrenergic CRC, we used a new publicly available data set from the Gabriella Miller Kids First (GMKF) Data Research Program (https://commonfund.nih.gov/kidsfirst/). This data set includes 124 tumors from low-, intermediate- and high-risk neuroblastoma cases (33), for which both rs2168101 genotypes and tumor cell RNA-Seq results are available. There were 7 neuroblastomas with *TATA/TATA* genotypes, which showed significantly lower *LMO1* expression levels than *GATA/TATA* or *GATA/GATA* tumors (*P <* 0.005; Figure 6A). It is notable that 6 of the 7 TATA/TATA tumors were classified as low risk (Figure 6A).

We next asked whether in human neuroblastoma, as in zebrafish neuroblastoma, the TATA/TATA genotype was significantly associated with usage of the mesenchymal CRC (Figure 5). For this analysis we applied a gene set signature score for every patient using adrenergic and mesenchymal signatures (6) and performed Gene Set Variation Analysis (GSVA). As in the zebrafish, we observed that none of the 7 human neuroblastomas with the *TATA/TATA* genotype had a positive adrenergic CRC signature score (Figure 6B). By contrast, neuroblastomas with *GATA/GATA* and *GATA/TATA* genotypes were approximately equally divided between those with predominately adrenergic and predominantly mesenchymal CRC signature scores (Figure 6, B and C). Within the *TATA/TATA* human tumors, the majority exhibited a positive score for the mesenchymal signature, which is consistent with our results in the zebrafish model (Figure 5A).

### Visualization of patterns of gene expression in primary human neuroblastomas.

To help visualize different subsets of neuroblastomas, we employed Uniform Manifold Approximation and Projection (UMAP) dimensionality reduction analysis based on the gene expression values for each tumor, then investigated how other features varied across the 2-dimensional UMAP embedding. We first analyzed the adrenergic signature score in the context of the UMAP values, which revealed 4 clusters of neuroblastomas (Figure 6D). Clusters 1d and 4d represent tumors with largely downregulated adrenergic signature scores (blue colored density contours), and clusters 2d and 3d represent tumors with largely upregulated adrenergic signature scores (red colored density contours) (Figure 6D). By contrast, the mesenchymal signature in Figure 6E is represented largely by a reciprocal pattern, in that it is upregulated within tumors mapped by contours corresponding to cluster 1e, while it is downregulated in tumors falling within the contours of clusters 2e to 4e. Interestingly, cohort 4d tumors not only have low adrenergic signature scores, but also have low mesenchymal signature scores, possibly indicating that they are driven by an alternative CRC with a different signature (34).

In Figure 6F, showing the UMAP coordinates grouped by *LMO1* expression levels, the tumors with low *LMO1* expression levels in cluster 1f correspond to a subset of the tumors with low adrenergic scores (cluster 1d; Figure 6D) and high mesenchymal scores (cluster 1e; Figure 6E). This cluster contains 6 of the 7 TATA/TATA tumors, which is consistent with their low *LMO1* expression levels. Interestingly, tumors within cluster 2f in Figure 6F also have low *LMO1* expression levels but can adopt the adrenergic signature (cluster 1d; Figure 6D). To determine whether this apparent discrepancy might be due to high levels of expression of *LMO* family members other than *LMO1*, we assessed *LMO2* and *LMO3* expression levels across the UMAP coordinates (Figure 6, G and H). *LMO2* is very low overall, as shown in Figure 3B, and, except for rare cases with moderate *LMO2* expression, the contours representing *LMO2* expression in Figure 6G do not reach levels at which *LMO2* could substitute for *LMO1*. This is consistent with other studies in mice implicating *lmo1* and *lmo3* as the closely related LMO family members normally expressed by neuronal cells (25). Higher levels of *LMO3* expression occurred in some of the human neuroblastomas corresponding to cluster 2h based on the adrenergic score (Figure 6H), explaining how the adrenergic signature can form in these tumors despite relatively low levels of *LMO1* expression. Interestingly, the human neuroblastomas expressing high levels of *LMO3* in cluster 2h are enriched for neuroblastomas with *MYCN* gene amplification (Figure 6I, cluster 2i). Tumors mapped mainly to cluster 3h have high *LMO1* expression levels (cluster 3f; Figure 6F) and have high adrenergic signature scores and low mesenchymal signature scores (cluster 3d and 3e in Figure 6, D and E).

Thus, detailed gene expression studies in both zebrafish and human neuroblastomas reveal conservation of tumor cell–specific gene expression signatures driven by the adrenergic and mesenchymal CRCs. The role of LMO1 as an essential cofactor for the adrenergic subtype of neuroblastoma appears to have been highly conserved throughout the 400 million years of evolution that separate zebrafish and human populations.

## Discussion

Many SNPs tightly linked to disease phenotypes by GWAS are located within noncoding regions of the genome, implying that they affect regulatory sequences and alter the expression of genes rather than the structure of the encoded proteins (35–38). Functional in vivo analysis of SNPs implicated by GWAS, so far, has generally not included the introduction of the orthologous base change in the noncoding genome, but rather has mainly focused on in vitro reporter assays of gene expression, epigenetic analysis of the associated gene locus, and in vivo knockout models of the implicated gene (39–41). However, studying the precise regulatory SNPs in animal models, as we have done here, is of critical importance to identifying the context and causal mechanisms that regulate the expression levels of disease-associated genes. One example of the usefulness of in vivo models for study of SNPs implicated by GWAS is the work of Madelaine et al. (42). Their work shows that deletion of highly conserved, noncoding DNA sequences, including a SNP linked to retinal vasculature defects, did not result in downregulation of MEF2C, the original candidate gene associated with this disease. Instead, the deletion of this region caused downregulation of expression of the miRNA-9 gene, and programmed depletion of this microRNA reproduced the retinal vasculature phenotype (42). Thus, the use of genomic studies in the zebrafish animal model have helped to clarify mechanisms underlying genetic loci implicated by GWAS (43–45).

### The regulatory SNP rs2168101 predicts neuroblastoma risk in zebrafish as well as human populations.

Here, we studied the impact and mechanism of rs216810, one of the SNPs most significantly associated with the risk of developing neuroblastoma in children. We first noted that WT zebrafish only have the G-containing allele at the nucleotide corresponding to the G/T SNP at rs2168101 in human populations. This was expected, because the G-containing allele is conserved throughout vertebrate evolution, and the T-containing allele polymorphism at this position arose first in human populations (Figure 1). Hence, we used genome editing technology to introduce the T-containing allele into the zebrafish germline and then bred this allele into our *MYCN* driven neuroblastoma model. We found significantly reduced penetrance of MYCN-driven neuroblastoma in transgenic zebrafish with the T/T allele at the rs2168101 locus (Figure 2), concurrent with decreased *lmo1* expression by these tumors (Figure 3), supporting our previous studies in neuroblastoma cell lines (20). Our studies in the zebrafish model, therefore, support a causative role for the G-containing allele at rs2168101 in upregulating *lmo1* expression levels in developing neuroblasts, which increases the rate of initiation of *MYCN*-driven neuroblastoma in this model (20). However, while we did demonstrate that GATA3 does not bind to the TATA allele at rs2168101 in human neuroblastoma cell lines (20), our findings in the zebrafish did not directly demonstrate that GATA3 could not bind to the TATA sequence at this locus. This was due to difficulties involved in performing ChIP-Seq with the small numbers of tumor cells available from zebrafish TATA/TATA tumors. Thus, we cannot rule out that the TATA knockin at this locus in the zebrafish may interfere with binding of other transcription factors as well, including other transcription factors within the adrenergic CRC, which could contribute to the decreased tumor penetrance (Figure 2) and loss of adrenergic CRC expression (Figure 4). It is also possible that the decreased tumor initiation of neuroblastoma in *lmo1^–/–^* or *TATA/TATA* zebrafish in our MYCN-driven neuroblastoma model may reflect a role of *lmo1* function in normal development of the PSNS. Thus, when *lmo1* expression is low or absent, hypoplasia of the sympathoadrenal cells may occur within the intrarenal gland during early development, thus decreasing the numbers of cells susceptible to MYCN-induced transformation. Further study will be required to investigate this possibility in our zebrafish models.

### The CRC itself determines the frequency and penetrance of neuroblastoma.

The central role and conservation of tissue-specific CRCs in development and cancer is supported by our finding that neuroblastomas arising in WT GATA/GATA fish overexpress genes typical of the adrenergic CRC (Figure 4B). In fish, as in human neuroblastomas, this permissive SNP drives high levels of expression LMO1, which is an essential transcriptional cofactor of the adrenergic CRC (13). By contrast, fish with the TATA/TATA allele specifically downregulate adrenergic CRC genes and instead exhibit high levels of expression of genes associated with the mesenchymal CRC (Figure 5A). Analogous to human neuroblastomas, the mesenchymal subtype of neuroblastoma in TATA/TATA zebrafish neuroblastomas is associated with activation of *hes2, jag1,* and *notch2,* indicating activation of the NOTCH signaling pathway (Figure 5C). This finding agrees with work of van Groningen and coworkers showing that activation of NOTCH signaling participates in the induction of the mesenchymal cell state in human neuroblastomas (10). In our study, we used cell sorting based on *dβh*-driven EGFP expression to direct our RNA-Seq studies to the *dβh*-expressing, EGFP^+^ neuroblastoma cells. This has the advantage of focusing the analysis on the tumor cell population, but it also has the disadvantage of excluding stromal cells of the tumor niche and infiltrating blood cell populations that may collaborate in unique ways in the tumor environments developing in individuals with different germline genotypes. Due to the decreased occurrence, slow growth rate, and small size of neuroblastomas in the TATA/TATA zebrafish, only 2 sets of RNA-Seq results were obtained for the transcriptome profiling of tumors in the TATA/TATA zebrafish. Future studies are warranted to capitalize on newer single-cell RNA-Seq technologies of unsorted cells from the tumors in these fish; newer technology would allow for simultaneous analysis of gene expression of EGFP^+^ cells and gene expression of the many other types of cells in the tumor niche that participate in the formation of MYCN–driven neuroblastoma.

### The TATA/TATA genotype at rs2168101 favors the onset of neuroblastomas with mesenchymal rather than adrenergic CRCs.

Our current study also includes what we believe to be new information from a new genome DNA and RNA sequencing data set by 124 primary childhood neuroblastomas, created with support of the Gabriella Miller Kid’s First Pediatric Research Program (Figure 6). This data set is particularly important because in earlier studies we used the TARGET data set, which was limited to tumors from children with high-risk neuroblastoma.

Our finding that the relatively infrequent tumors that arise in the TATA/TATA zebrafish exhibit the mesenchymal CRC signature is consistent with our previous studies showing that a high level of LMO1 expression is required for the adrenergic CRC to form in human neuroblastoma (13). In agreement with these findings in the TATA/TATA zebrafish, our data using the Gabriella Miller human neuroblastoma data set also shows that human tumors arising in children with the TATA/TATA genotype at rs2168101 exhibit the mesenchymal signature, and most of these tumors are assigned to children with low-risk, localized neuroblastomas that are often cured by surgery alone (Figure 6). Thus, the TATA/TATA genotype provides a reassuring biomarker associated with nonmetastatic disease for children undergoing treatment. Unfortunately, the genotype of rs2168101 does not signify sensitivity to a targeted drug at this time, as would be indicated, for example, by an activating ALK mutation.

Moreover, we can anticipate how the *TATA/TATA* genotype would influence the penetrance of neuroblastoma in children who also have germline predisposition due to activating mutations of *PHOX2B* or *ALK*. Because the TATA/TATA genotype causes low *LMO1* expression and *LMO1* is required for the adrenergic CRC to form, we predict that the *TATA/TATA* genotype would protect against neuroblastoma in patients with germline activating mutations of *PHOX2B* (46, 47). This is because PHOX2B is an essential driver of the adrenergic CRC, which cannot form efficiently without high-level expression of LMO1. Less is known about the impact of *ALK* mutations on the CRC in neuroblastoma, but most available neuroblastoma cell lines that harbor activating *ALK* mutations, such as Kelly and SY5Y, rely on the adrenergic CRC. Thus, it appears likely that the *TATA/TATA* genotype would also decrease the penetrance of neuroblastoma in children harboring germline activating *ALK* mutations. Further study will be required to test these predictions.

An apparent paradox emerges, however, because others have shown that GATA/GATA or GATA/TATA neuroblastoma may shift from the adrenergic to the mesenchymal cell state under the selection pressure of chemotherapy, presumably because the mesenchymal cell state imparts drug resistance (6, 48). However, this represents an apparent paradox, because the mesenchymal cell state appears to be associated both with the initiation of localized tumors in young children and with rapidly growing and metastatic drug resistant neuroblastomas that are not responding to therapy. To explain this apparent discrepancy, we hypothesize that adrenergic neuroblastoma cells can both switch to the mesenchymal cell state and select for RAS-MAPK pathway mutations under the pressure of intensive combination chemotherapy (6, 11, 48–52). Thus, the switch to the mesenchymal cell state provides immediate resistance to antineuroblastoma drugs but relatively indolent cell growth, allowing for the long-term outgrowth of rare subclones of cells with drug resistance due to RAS-MAPK pathway mutations. These mutations impart aggressive proliferative and metastatic growth properties and may allow neuroblastoma cells to shift back to the adrenergic cell state. We have already shown that homozygous *NF1* inactivating mutations confer very aggressive growth properties in the zebrafish neuroblastoma model (53). RAS-MAPK mutations are not present in low-risk TATA/TATA tumors that arise de novo with the mesenchymal cell state, thus, these neuroblastomas remain localized and are treatable with surgery alone. The hypothesis that neuroblastoma cells harbor both RAS-MAPK pathway mutations and the adrenergic pattern of gene expression at relapse after intensive chemotherapy regimens could be tested if both whole-genome DNA-Seq and genome-wide RNA-Seq studies were available from cohorts of patients treated with intensive chemotherapy.

Our results also revealed that a subset of the GATA/TATA neuroblastomas with lower LMO1 expression can develop in neuroblastomas with the adrenergic CRC, due, in part, to aberrant upregulation of LMO3, a closely related LMO family member. This is the case for the neuroblastoma tumors in our UMAP analysis (cluster 2h, Figure 6H), which are characterized by lower *LMO1* but high *LMO3* expression levels (Figure 6, F and H). These tumors are enriched for cases with a heterozygous *GATA/TATA* genotype, and elevated *LMO3* levels apparently cooperate with *LMO1* to drive the adrenergic CRC. The redundancy of *LMO* gene family members is analogous to the findings in the TAL1-overexpressing subtype of T-ALL, in which *LMO1* or *LMO3* can be aberrantly activated by chromosomal translocation and substitute for *LMO2* in the TAL1 CRC that drives thymocyte transformation (54–56). In this regard, it is interesting that aberrantly high *LMO3* expression levels appear to be frequent in neuroblastomas with *MYCN* gene amplification (Figure 6I), which may explain the lack of association of the rs2168101 genotype with the risk of developing *MYCN*-amplified neuroblastomas (20).

### lmo1^–/–^ zebrafish do not exhibit either the mesenchymal or adrenergic CRC.

An unexpected finding arising from our in vivo zebrafish model is that neuroblastomas arising in the *lmo1*^–/–^ background expressed genes that were not associated with either the adrenergic or the mesenchymal CRC, but instead showed upregulation of a third set of transcription factors (Figure 5D). Our hypothesis is that, by preventing high levels of lmo1 expression in neuroblastoma, the *TATA/TA*TA genotype blocks the formation of the adrenergic CRC because this CRC requires high levels of the lmo1 cofactor. The use of the mesenchymal CRC by these tumors represents the well-published tendency of neuroblastomas to shift to the mesenchymal CRC when use of the adrenergic CRC becomes problematic. In the *lmo1^–/–^* tumors, a different CRC may arise because this genotype also blocks the mesenchymal CRC from forming. This could be due to the loss of *lmo1* expression by noncell-autonomous stromal cells, which would happen in a complete *lmo1*-knockout animal. However, stromal *lmo1* expression would be expected to remain intact to support the notch-dependent mesenchymal CRC in *TATA/TATA* neuroblastoma. This is supported by the fact that the *lmo1^–/–^* tumor cells fail to show signs of Notch signaling, which is very important to induce the mesenchymal CRC (10). Notch signaling is also an important candidate pathway to play a noncell-autonomous role in neuroblastoma initiation, because NOTCH signaling is the prototypic pathway demonstrating the essential role of the signaling cell as well as the receiving cell for successful signal transduction (57, 58). The tissue-specific nature of CRCs and enhancers is illustrated by our finding that *LMO1* expression is known to be regulated in human tumors by different CRCs with different enhancers in specific tissues. For example, the super-enhancer surrounding rs2168101 is not formed in T-ALLs expressing high levels of *LMO1*, but, rather, high levels of *LMO1* are driven by completely different enhancers. Other possibilities suggested to explain the different CRCs found in *lmo1^–/–^* and *TATA/TATA* neuroblastomas include, first, differential effects on early PSNS development of *lmo1* knockout compared with mutations creating *TATA/TATA* genotypes, or, second, possible effects of the mutations we introduced to produce the *TATA/TATA* genotype in fish on the binding of other CRC transcription factors. Thus, in our future studies, it will be important to conduct single-cell RNA-Seq of unsorted cells from the neuroblastoma as they develop in fish with the different rs2168101 and *lmo1* genotypes in order to test the hypothesis that the TATA/TATA genotype primarily reduces *lmo1* expression in neuronal progenitor cells, while the *lmo1* knockout affects lmo1-regulated gene expression in every tissue, including adrenal medullary stromal cells.

### Conclusions.

Overall, our studies support the value of zebrafish and other in vivo model systems to investigate the mechanisms underlying the multi-step process of clonal progression in the initiation of cancers like neuroblastoma*,* and to link these underlying mechanisms to GWAS-based associations with key haplotypes that predict the risk of developing specific types of human cancers.

## Methods

### Zebrafish lines and maintenance.

Zebrafish were derived from the AB background strain. All zebrafish were maintained under standard aquaculture conditions at the Dana-Farber Cancer Institute. Zebrafish lines *Tg(dβh:EGFP)* and *Tg(dβh:MYCN)* were described previously (24) and designated *EGFP* and *MYCN* in the text, respectively.

### Genome editing of lmo1 using TALEN and CRISPR/Cas9 technology.

To generate stable lines carrying the protective T-containing allele at rs2168101 in the first intron of *lmo1*, we designed TALEN-recognition sequences that bind to the first intron of *lmo1* surrounding the *GATA* site: TALEN1 5′-TACGACTGATTTGATTTT-3′ and TALEN2 5′-TTCATTTCAAGTTCCAT-3′. A 16-bp spacer with an EcoRV sequence was located between the 2 binding sites. TALEN expression vectors harboring a WT FokI nuclease were generated as previously described (59), linearized by PmeI and used as templates for TALEN mRNA synthesis using the mMessage mMachine T3 Kit (Ambion). For the targeted integration of the protective T-containing allele, we generated a 41-nucleotide single-stranded oligonucleotide (*TATA-ssOligo*) containing the T-allele sequence with 20 flanking nucleotides on either side of the T. The *TATA-ssOligo* also contains 2 additional nucleotide changes that are located 5′ to the T (CC instead TT; Figure 2A) to not only create a new binding site for TfiI (to allow for screening for offspring with successful knock-in of the *TATA-ssOligo*) but also to prevent the TALEN1 from binding and cutting out the integrated oligonucleotide. Equal amounts of TALEN1 and 2 mRNA (100 ng/μL) together with 100 pg of oligonucleotide were injected together into one-cell–stage zebrafish embryos. To identify positive founder fish with a successful TATA knock-in, germline DNA was extracted by tail clip, and the DNA fragment surrounding the TATA site was amplified using the primers (*TATA/TATA-EcoRV*) listed in Supplemental Table 1, followed by EcoRV digestion. Successful genome editing was detectable by loss of the EcoRV restriction site (Supplemental Figure 3A). Only fish incorporating the *TATA-ssOligo* screened positive in the TfiI assay, where TfiI digestion of an amplicon generated using the primers (*TATA/TATA-TfiI*) listed in Supplemental Table 1 resulted in 2 DNA fragments: an undigested 130-bp long fragment and a digested 65-bp long fragment (Supplemental Figure 3B). Using this approach, we isolated a zebrafish line with a heterozygous T-containing allele at rs2168101 (designated “*TATA/GATA*”, Figure 2A). F1 embryos at 24 hours postfertilization (hpf) were again screened for germ-line transmission, and the F1 embryos from the positive founder were raised to adulthood. The F2 generation was crossed with the zebrafish lines *Tg(dβh:EGFP)* and *Tg (dβh:MYCN)* and then intercrossed to obtain F4 heterozygous, homozygous mutant offspring, and WT *(GATA/GATA*) offspring in the *dβh:MYCN;dβh:EGFP* double transgenic background.

The *lmo1* knockout zebrafish line (*lmo1*^–/–^) was generated by using CRISPR-Cas9 genome editing technology, targeting exon 2 (60). The following *lmo1* exon 2 CRISPR site was designed using a CRISPR Design web tool (http://crispr.mit.edu): 5′-GGAGAGGGAGATCAGATCGA-3′, CRISPR gRNA was prepared by the cloning-free, single-guide RNA synthesis method (61) using the Ambion T7 MEGAscript Kit (Ambion) and purified with the Qiagen miRNeasy Kit (Qiagen). *Cas9* nuclease was purchased from New England Biolabs. Each embryo was injected with 1 nl of solution containing *lmo1*-specific gRNA (60 ng/μl) and Cas9 (30 ng/μl) at the one-cell stage. Mosaic F0 fish with germline mutations were identified, and the stable mutant line for *lmo1^+/–^* was established by outcrossing to WT AB fish. To genotype the *lmo1^+/–^* line 3 months after injection, genomic DNA was extracted from fin clips, and DNA fragments containing the mutated site were amplified using the primers (*lmo1*^–/–^) listed in Supplemental Table 1. The amplified DNA product was analyzed using the T7 endonuclease I mismatch cleavage assay to detect mutations introduced after Cas9 cleavage. One line was identified with a 32-nucleotide deletion starting at coding nucleotide 165 that caused an early stop codon at the end of the first LIM domain and a complete loss of the second LIM domain (Supplemental Figure 4). This line, therefore, harbors a loss-of-function allele of *lmo1* (*lmo1*^+/–^). The F2 zebrafish were crossed with the zebrafish lines *Tg(dβh:EGFP)* and *Tg (dβh:MYCN)*, and next intercrossed to obtain F4 *lmo1*^+/+^, *lmo1*^+/–^, and *lmo1*^–/–^ offspring in the *dβh:MYCN;dβh:EGFP* double-transgenic background.

### Neuroblastoma tumor watch.

*Lmo1^+/–^* and *TATA/GATA* zebrafish were crossed with *dβh:MYCN^+^* and *dβh:EGFP^+^* zebrafish to obtain heterozygous *lmo1^+/–^* and *GATA/TATA* lines in each transgenic background. These offspring were sorted for EGFP^+^ fluorescence and genotyped for *TATA/GATA* or *lmo1^+/–^* heterozygosity and inbred to their siblings to obtain all possible genotypes: *GATA/GATA* (WT), *TATA/GATA*, and *TATA/TATA,* or *lmo1^+/+^*, *lmo1^+/–^*, and *lmo1^–/–^* in the *dβh:MYCN;dβh:EGFP* double-transgenic background. Clutches of growing progeny from heterozygous *GATA/TATA* or *lmo1^+/–^* incrosses were examined weekly to identify EGFP^+^ tumors as they arose in the different genotypes of growing zebrafish. Trained zebrafish researchers anesthetized fish from each incross with tricaine and carefully examined them under a fluorescence microscope every 2 weeks starting at week 7 to identify any fish that developed a visible EGFP-fluorescent group of cells in the position of the IRG. Until week 7, the IRG is often transiently visible by EGFP fluorescence, therefore zebrafish are not routinely examined for tumor masses before week 7. Fish with visible EGFP^+^ cells from each week of life after week 7 from each clutch were separated and grown together in a separate tank. To make sure that the identified EGFP^+^ cells represented a growing tumor, each of the fish with EGFP^+^ fluorescence was also reexamined during each subsequent week to make sure the EGFP^+^ cells were growing in size, indicating a transformed tumor cell population. In the rare case that the EGFP^+^ cells in a fish regressed after being identified after 7 weeks of age, then this fish was removed and placed back with the negative fish from the clutch. In the fish, in which an EGFP^+^ tumor continued to grow, the date of onset was designated as the date on which the fish was first identified and grown in a separate tank due to detection of an EGFP^+^ mass. The fish with EGFP^+^ tumors were then grown together until the tumors are large enough to harvest, and then the EGFP^+^ cells were harvested for flow sorting and RNA extraction. At the same time, these fish were genotyped DNA extracted from a fin clip to determine whether they were *GATA/GATA*, *GATA/TATA* or *TATA/TATA*. Thus, the tumor watch was done blind to the genotype of the fish in each clutch. At the end of the experiment at 17 weeks of age, fish from each clutch were genotyped to establish the tumor incidence for fish of each genotype. The cumulative frequency of neuroblastoma development was analyzed by the Kaplan-Meier method, and the log-rank test was used to determine statistical significance.

### RNA-Seq.

Total RNA was extracted from neuroblastomas arising in *TATA/TATA* and *lmo1*^–/–^ in the *MYCN;EGFP* background using the QIAzol lysis reagent (Qiagen) and was cleaned using the RNeasy kit (Qiagen). Samples were treated with the TURBO DNase (TURBO DNA-free Kit; Ambion) and cleaned using the RNeasy MinElute Cleanup kit (Qiagen). Strand-specific library construction and Illumina HiSeq sequencing of paired-end 100-bp-long reads were performed at the DFCI Molecular Biology Core Facilities. Newly generated RNA-Seq data were combined with previously generated data sets (62). Normalized RPKM files (MYCNoe) were downloaded from GSE107518 and used as GATA/GATA. For samples newly generated herein, RNA-Seq reads were aligned to the danRer10 revision of the zebrafish reference genome using hisat2 version 2.1.0 (63) in paired-end mode. Reads in v90 of GRCz10 Ensembl genes were quantified using htseq-count (64) with parameters -i gene_name —stranded=reverse -m intersection-strict. Expression as reads normalized by kb of exon per million mapped reads (RPKM) were calculated by counting the number of nonredundant basepairs in all isoforms of each gene with the same gene name. All RNA-Seq data have been deposited in the GEO database (GSE224158).

### Gene set variation analysis.

GSVA was performed in R (version 4.0.2) using the GSVA library (version 1.38.2). For each gene set of interest, scores were computed using the “kcdf=Poisson” setting with raw read counts from the Gabriella Miller Kids First neuroblastoma RNA-Seq cohort as input. Specifically, adrenergic and mesenchymal pathway activation scores were calculated using gene lists as previously defined by Van Groningen et al. (6).

### UMAP analysis.

UMAP analysis was performed in R (version 4.0.2) using the Seurat library (version 4.0.0). Raw fragments per kilobase of transcript per million mapped reads (FPKM) data were first log_2_-transformed and scaled using default Seurat parameters. Principal components were then computed and scored using the jackstraw procedure. Ten principal components (corresponding to all components with nominal jackstraw *P* < 0.05) were then provided as input for 2-dimensional UMAP embedding. To overlay quantitative features including GSVA pathway activation scores or log_2_-transformed FPKM gene expression scores, we first computed the Z-score transform of the corresponding feature. To compute contour plots over the UMAP embedding space, we applied a Z-score-weighted Gaussian kernel to each patient datapoint that was radially symmetric with a corresponding variance equal to one half.

### rs2168101 evolution and conservation analysis.

Allelic frequencies of rs2168101 across human populations were obtained from the 1,000 Genomes Project Phase 3 data (http://useast.ensembl.org/Homo_sapiens/Variation/Population?v=rs2168101). Multiple species alignment of 40 reference genomes surrounding the rs2168101 GATA site were made based on publicly available data sets on ENSEMBLE and the UCSD Genome Browser. A corresponding phylogenetic tree based on these cross-species sequences was created using the publicly available source https://www.phylogeny.fr (65).

### Adrenergic and mesenchymal gene signatures and gene ranking for Figure 4C.

Signatures corresponding to adrenergic (*n* = 369) or mesenchymal genes (*n* = 485) were obtained from Van Groningen, et al. (6). mRNA expression (TPM+1) of each gene in these signatures was obtained for each neuroblastoma cell line from the Cancer Cell Line Encyclopedia (22Q2 release). Median adrenergic and mesenchymal gene scores were calculated for each cell line by rank ordering all genes based on expression level in the combined signatures (*n* = 854) from lowest to highest expression, such that rank 854 represented the highest expressed gene in the signature. Gene ranks were then separated into adrenergic (*n* = 369) and mesenchymal (*n* = 485) lists, and the median rank score was determined for each signature separately, to determine whether an individual cell line predominately exhibited the adrenergic or mesenchymal cell state.

### Data and materials availability.

All RNA-Seq data have been deposited in the GEO database (GSE No: pending GSE224158).

Neuroblastoma RNA-Seq available through the Gabriella Miller Kids First (GMKF) Pediatric Research Program: https://commonfund.nih.gov/kidsfirst.

### Statistics.

Statistical analysis was performed using GraphPad Prism software version 8.0. Kaplan-Meier curves and log-rank tests were used to assess the rate of tumor development and differences in the cumulative frequency of neuroblastoma between fish with the following genotypes: *dβh:MYCN; dβh:EGFP*, *lmo1^+/+^*, *dβh:MYCN; dβh:EGFP*, *lmo1*^+/–^, *dβh:MYCN; dβh:EGFP*, *lmo1*^–/–^, *dβh:MYCN; dβh:EGFP*, *TATA/GATA*, and *dβh:MYCN; dβh:EGFP*, *TATA/TATA*. Significance was determined using unpaired, 2-tailed *t* tests, Student’s *t* tests, and Welch’s *t* tests. *P* < 0.05 was considered significant. For all experiments with error bars, the SEM was calculated, and the data were presented as mean ± SD. The sample size for each experiment and the replicate number of experiments were included in the figure legends.

### Study approval.

All experiments were approved by the Dana-Farber IACUC under protocol no. 02-107.

## Author contributions

NWL, JMM, and ATL conceived the study and designed the experiments. NWL, TT, HS, SZ, ADD, and MWZ performed zebrafish experiments and gene expression assays. DAO, ACW, and SJD analyzed human gene expression assays. DAO performed the phylogenetic tree analysis and the analysis of the human neuroblastoma gene expression data. DR and JKJ created the TALENs used in this study. BJA and RAY performed the analysis of zebrafish gene expression. NWL and ATL wrote the manuscript with input from each of the authors.

## Supplementary Material

Supplemental data

## Version 1 05/15/2023

Electronic publication
