# Supplementary material for: Genetic predisposition to neuroblastoma results from a regulatory polymorphism that promotes the adrenergic cell state
Source: J Clin Invest. 2023 May 15;133(10):e166919. doi: 10.1172/JCI166919 (PMC10178836; doi:10.1172/JCI166919)
Supplement: Supplemental data [file jci-133-166919-s191.pdf]

Supplemental Material

Genetic Predisposition to Neuroblastoma Results from a Regulatory Polymorphism that Promotes the Adrenergic Cell State

Authors: Nina Weichert-Leahey [1,2], Hui Shi [1,3], Ting Tao [1,4,5], Derek A. Oldridge [6], Adam D. Durbin [7], Brian J. Abraham [8,9], Mark W. Zimmerman [1], Shizhen Zhu [10], Andrew C. Wood [11], Deepak Reyon [12,13], J. Keith Joung [12,13], Richard A. Young [8,14], Sharon J. Diskin [15], John M. Maris [15]*, A. Thomas Look[1, 2]*.

Supplemental Figures

Supplemental Figure 1

```
Zebrafish                AAA-GGGTAC-GACTGATTTGATTTTTATGATATCCACAAAAATGGAACTTGAAATGAAG
Zig-zag eel              AAAAGTTAGCAGACTGATGTGATTTTTTTGATAGCTGTAAAGACGGAAGTCTAAAT--GG
Spotted gar              AAAGGGTTACCACGTTGATTTGATTTTTCGATAGCTGTAAAAATGGAACTCGAAAT--GG
Red-bellied piranha      AAA-GGGTACCATGTTGATTTGATTTTTTGATAGCTGCAAAAATGGAACTTGAAATGGGG
Opossum                  AAAGGTTTGCAGGGCAGGCTTGATTTTTAGATAACTCTAAAGTGGAT-------CCCAAA
Coelacanth               AAAGGATTATAGTGCACATTTGATTATTAGATAGTGCTGAAATGGAT-------CCCAAA
Anole lizard             AAAGGATTACCGTGTAGATTTGATTTTTAGATAACACTAAAATGGAT-------CTCAAA
Turkey                   AAAGGATTACCATGTAGATTTGATTTTTAGATAACACTAAAATGGAT-------CCCAAA
Zebra Finch              AAAGGATTACCATGTAGATTTGATTTTTAGATAACACTAAAATGGAT-------CCCAAA
Rat                      AAAGGATTACCATGTAGATTTGATTTTTAGATAACGCTAAAATGGAT-------CCCAAA
Ryukyu mouse             AAAGGATTACCATGTAGATTTGATTTTTAGATAACGCTAAAATGGAT-------CCCAAA
Shrew mouse              AAAGGATTACCATGTAGATTTGATTTTTAGATAACGCTAAAATGGAT-------CCCAAA
Mouse                    AAAGGATTACCATGTAGATTTGATTTTTAGATAACGCTAAAATGGAT-------CCCAAA
Algerian mouse           AAAGGATTACCATGTAGATTTGATTTTTAGATAACGCTAAAATGGAT-------CCCAAA
Chinese hamster          AAAGGATTACCATGTAGATTTGATTTTTAGATAACACTAAAATGGAT-------CCCAAA
Prairie vole             AAAGGATTACCATGTAGATTTGATTTTTAGATAACGCTAAAATGGAT-------CCCAAA
Gibbon                   AAAGGATTACCATGTAGATTTGATTTTTAGATAACACTAAAATGGAT-------CCCAAA
Elephant                 AAAGGATTACCATGTAGATTTGATTTTTAGATAACACTAAAATGGAT-------CCCAAA
Guinea Pig               AAAGGATTACCATGTAGATTTGATTTTTAGATAACACTAAAATGGAT-------CCCAAA
Horse                    AAAGGATTACCATGTAGATTTGATTTTTAGATAACACTAAAATGGAT-------CCCAAA
Marmoset                 AAAGGATTACCATGTAGATTTGATTTTTAGATAACACTAAAATGGAT-------CCCAAA
Bonobo                   AAAGGATTACCATGTAGATTTGATTTTTAGATAACACTAAAATGGAT-------CCCAAA
Pig                      AAAGGATTACCATGTAGATTTGATTTTTAGATAACACTAAAATGGAG-------CCCAAA
Sheep                    AAAGGATTACCATGTAGATTTGATTTTTAGATAACACTAAAATGGAT-------CCCAAA
Dog                      AAAGGATTACCATGTAGATTTGATTTTTAGATAACACTAAAATGGAT-------CCCAAA
Dingo                    AAAGGATTACCATGTAGATTTGATTTTTAGATAACACTAAAATGGAT-------CCCAAA
Cat                      AAAGGATTACCATGTAGATTTGATTTTTAGATAACACTAAAATGGAT-------CCCAAA
Leopard                  AAAGGATTACCATGTAGATTTGATTTTTAGATAACACTAAAATGGAT-------CCCAAA
Rabbit                   AAAGGATTACCATGTAGATTTGATTTTTAGATAACACTAAAATGGAT-------CCCAAA
Goat                     AAAGGATTACCATGTAGATTTGATTTTTAGATAACACTAAAATGGAT-------CCCAAA
Cow                      AAAGGATTACCATGTAGATTTGATTTTTAGATAACACTAAAATGGAT-------CCCAAA
Mouse Lemur              AAAGGATTACCATGTAGATTTGATTTTTAGATAACACTAAAATGGAT-------CCCAAA
Vervet-AGM               AAAGGATTACCATGTAGATTTGATTTTTAGATAACACTAAAATGGAT-------CCCAAA
Olive baboon             AAAGGATTACCATGTAGATTTGATTTTTAGATAACACTAAAATGGAT-------CCCAAA
Chimpanzee               AAAGGATTACCATGTAGATTTGATTTTTAGATAACACTAAAATGGAT-------CCCAAA
Gorilla                  AAAGGATTACCATGTAGATTTGATTTTTAGATAACACTAAAATGGAT-------CCCAAA
Crab-eating macaque      AAAGGATTACCATGTAGATTTGATTTTTAGATAACACTAAAATGGAT-------CCCAAA
Macaque                  AAAGGATTACCATGTAGATTTGATTTTTAGATAACACTAAAATGGAT-------CCCAAA
Orangutan                AAAGGATTACCATGTAGATTTGATTTTTAGATAACACTAAAATGGAT-------CCCAAA
Human                    AAAGGATTACCATGTAGATTTGATTTTTAGATAACACTAAAATGGAT-------CCCAAA
                         *** *              *   ** *  ****      **   *
```

Supplemental Figure 1: Sequence of nucleotides in the first intron of *LMO1* adjacent to the G allele at the rs2168101 in 40 species including zebrafish (on top) and human (on bottom). Grey box marks the *GATA* motif.

Supplemental Figure 2

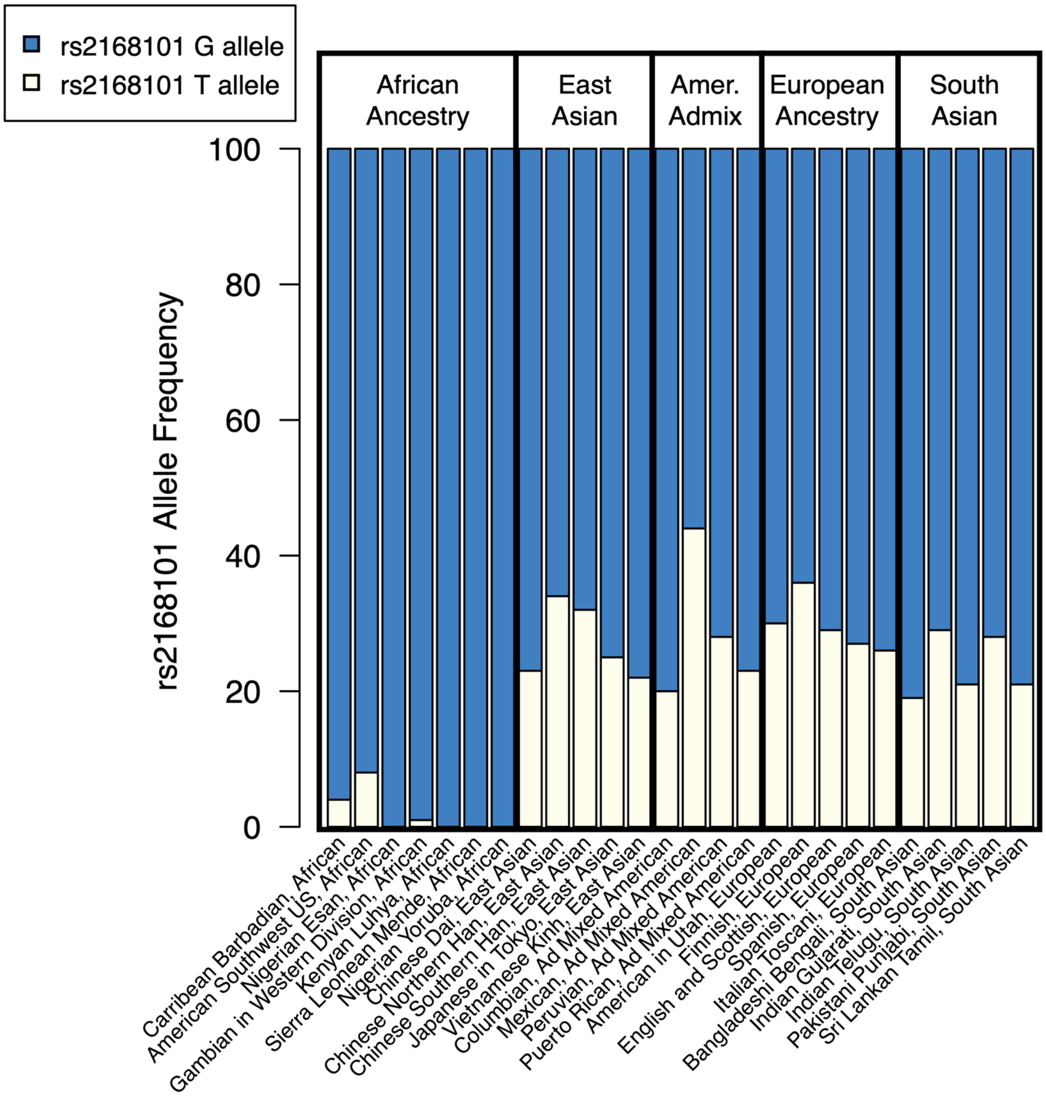

Supplemental Figure 2: Frequency of G and T alleles at rs2168101 within geographically defined human populations genotyped as part of the 1000 Genomes Project as indicated. Frequency of the G allele is marked in blue and of the T allele in white.

Supplemental Figure 3

A

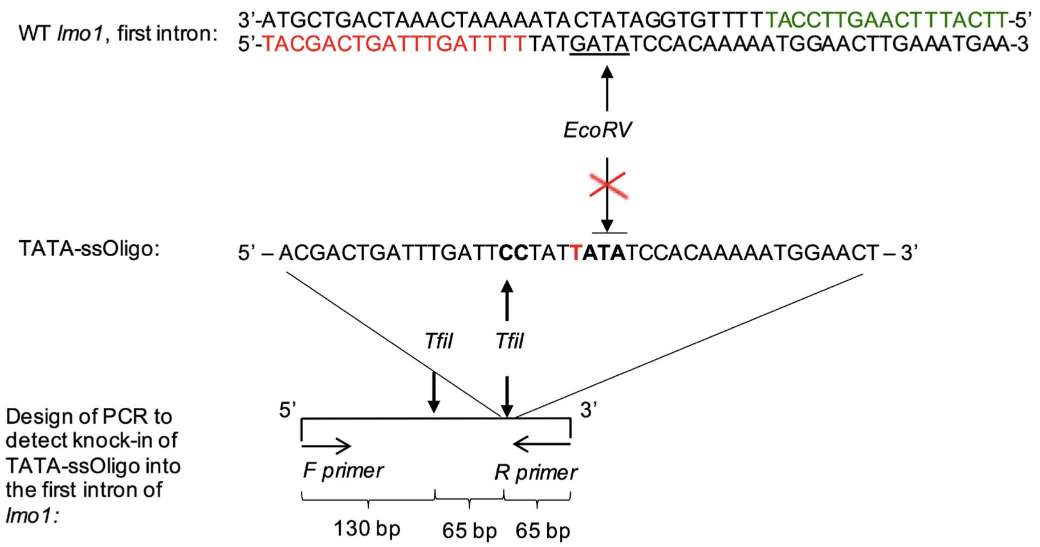

B

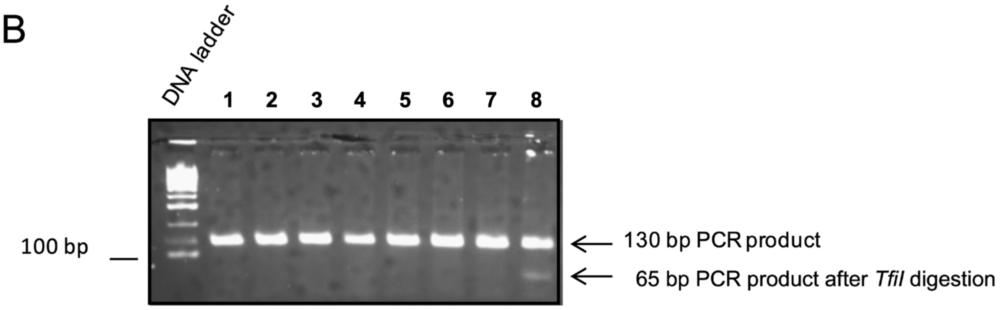

Supplemental Figure 3: Strategy for TALEN-mediated genome editing to introduce the rs2168101 T allele in zebrafish.

(A) Wild-type (WT) sequence of the first intron of *lmo1* flanking to the G allele at rs2168101 in zebrafish is shown. The TALEN binding sites are marked in red (TALEN1) and green (TALEN2), and the *GATA* site is underlined. The arrow points to the EcoRV restriction enzyme site that is present in the G allele but absent in the T allele. *TATA-ssOligo* is a 41-nucleotide, single-stranded DNA oligonucleotide that was co-injected with the sequence-specific TALENs into fertilized wild-

type zebrafish embryos at the one-cell stage of development to facilitate knock-in of the T at rs2168101 (marked in red). The *TATA-ssOligo* also includes two other nucleotide changes ("CC", marked in bold) on the 5′ side of the T, which were included to prevent unnecessary recurrent TALEN binding (see TALEN binding sites underlined above in the wild-type *lmo1* sequence) and to create a new TfiI restriction site for screening purposes. A PCR/Tfi1 digestion assay was designed to screen zebrafish embryos for knock-in of the *TATA* ss-Oligo into the first intron of *lmo1* (below).

(B) In the PCR/Tfi1 digestion assay, only embryos with successful knock-in of the rs2168101 T allele reveal a PCR product that can be Tfi1-digested into two 65-bp products.

Supplemental Figure 4

A

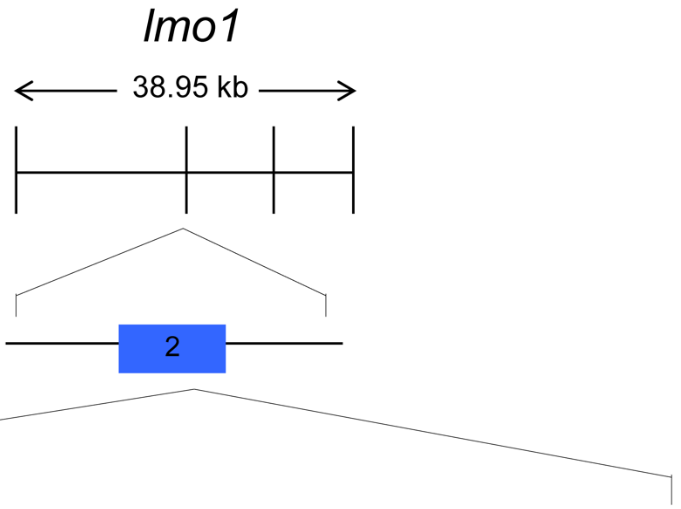

5' – GTGCCTGCTGTGACTG**CCG**CCTGGGGGAGGTGGGCTCCACCCTCTACACCAAAGCCAATCTCAT– 3'

B

lmo1 exon 2 ...TGACTG**CCG**CCTGGGGGAGGTGGGCTCCACCCTCTACACCAAAGCCAATCTCAT...

lmo1 Δ32 (-32) ...TGACTG**CC** - AAAAGCCAATCTCAT...

C

Lmo1
MVLDKEEGVPMLSVQPKGKQKGCAGCNRKIKDRYLLKALDKYWHEDCLKCACCDCRLGEVGSTLYTKANLIL
CRRDYLRLFGTTGNCAACSKLIPAFEMVMRARDNVYHLDCFACQLCNQRFCVGDKFFLKNNMILCQMDYEEG
QLNGSFETQVQ

Lmo1 Δ32
MVLDKEEGVPMLSVQPKGKQKGCAGCNRKIKDRYLLKALDKYWHEDCLKCACCDC***QSQSHPLSQGLPEAL***
WYNGELCSLQ**X**

D

Human LMO1 (158 a.a.)

Zebrafish Lmo1 (155 a.a.)

Zebrafish LMO1Δ32 (81 a.a.)

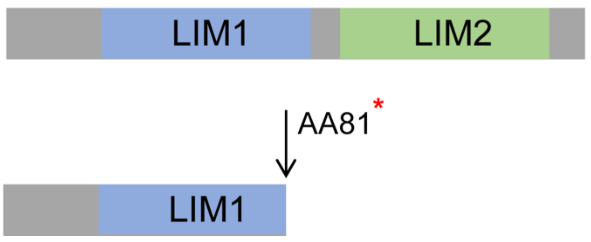

Supplemental Figure 4: Strategy for genome editing to disrupt the coding sequence of lmo1 and generate null alleles in zebrafish.

(A) A diagram of the *lmo1* gene is shown with expansion of its second exon below. The sequence recognized by the single guide RNA (sgRNA) in the coding sequence of *lmo1* exon 2 is marked in green and underlined, with the Cas9 recognition site marked in red.

(B) The WT sequence of the first intron of *lmo1* in zebrafish with the marked CRISPR/Cas9 site is shown in top panel. The sequence of the identified founder fish with a 32-nucleotide deletion ("lmo1 Δ32 (-32)") is shown below. The resulting knockout line is referred to as "lmo1-/-".

(C) The wild-type zebrafish Lmo1 protein sequence is shown above, with the corresponding protein sequence of the lmo1 Δ32 knockout mutant shown below with the early stop codon (red X).

(D) An illustration of the wild-type zebrafish Lmo1 protein is shown above with the LIM1 domain marked in blue and the LIM2 domain in green. The protein encoded by the lmo1 Δ32 mutant gene is shown below. The Δ32 deletion causes a frame shift and an early stop codon that truncates the first LIM domain and completely removes the second LIM domain.

Supplemental Table 1: Primers

zebrafish genes	Forward primer (5'-3')	Reverse primer (5'-3')
lmo1-/-	TTTCCAGACACAAGGACAAGGATCC	GCAGATATCAGAAGAAAGAGTACATTTGTCTT
TATA/TATA- Tfi1	GCTTTCAACTGTGTGCTCATTAAAAG	GATAGGATCCGGTTTCTATGTAGGC
TATA/TATA-EcoRV	GACCCTGCATACAGTAAAGTTTGCA	ATCCGCCTCTTTTCCGCCCATCCAC

Supplemental Table 2: *LMO1* expression in adrenergic and mesenchymal neuroblastoma cell lines

Cell Line	*LMO1* expression	Adrenergic/Mesenchymal
CHP126	4.13996057	Adrenergic
KPNRTBM1	4.660495132	Adrenergic
NH6	5.040015679	Adrenergic
COGN278	2.744161096	Adrenergic
CHLA15	5.21218021	Adrenergic
NB1643	2.077242999	Adrenergic
KPNYN	5.726831217	Adrenergic
SKNBE2	2.839959587	Adrenergic
MHHNB11	1.560714954	Adrenergic
Kelly	5.241077458	Adrenergic
SIMA	5.037821465	Adrenergic
IMR32	1.454175893	Adrenergic
LAN2	0.545968369	Adrenergic
COGN305	0.613531653	Adrenergic
NGP	3.160274831	Adrenergic
NMB	4.785027362	Adrenergic
SKNDZ	2.201633861	Adrenergic
SKNFI	6.355967689	Adrenergic
GIMEN	0	Mesenchymal
CHP212	0.678071905	Mesenchymal
SKNAS	1.137503524	Mesenchymal
KPNSI9S	0.443606651	Mesenchymal
SKNSH	3.894332742	Mesenchymal
